# Digital Microfluidic Multiplex RT-qPCR for SARS-CoV-2 Detection and Variants Discrimination

**DOI:** 10.3390/mi14081627

**Published:** 2023-08-17

**Authors:** Kuan-Lun Ho, Jing Ding, Jia-Shao Fan, Wai Ning Tiffany Tsui, Jianfa Bai, Shih-Kang Fan

**Affiliations:** 1 Department of Mechanical and Nuclear Engineering, Kansas State University, Manhattan, KS 66506, USA; kuanlun@ksu.edu (K.-L.H.); jingd@ksu.edu (J.D.); 2 Department of Electrical and Computer Engineering, Kansas State University, Manhattan, KS 66506, USA; joshuafan@ksu.edu; 3 Kansas State Veterinary Diagnostic Laboratory, Kansas State University, Manhattan, KS 66506, USA; wntsui@vet.ksu.edu (W.N.T.T.); jbai@vet.ksu.edu (J.B.); 4 Department of Diagnostic Medicine/Pathobiology, Kansas State University, Manhattan, KS 66506, USA

**Keywords:** SARS-CoV-2, COVID-19, Delta variant, Omicron variant, RT-qPCR, digital microfluidics, electrowetting

## Abstract

Continuous mutations have occurred in the genome of the SARS-CoV-2 virus since the onset of the COVID-19 pandemic. The increased transmissibility of the mutated viruses has not only imposed medical burdens but also prolonged the duration of the pandemic. A point-of-care (POC) platform that provides multitarget detection will help to track and reduce disease transmissions. Here we detected and discriminated three genotypes of SARS-CoV-2, including the wildtype and two variants of concern (VOCs), the Delta variant and Omicron variant, through reverse transcription quantitative polymerase chain reaction (RT-qPCR) on a digital microfluidics (DMF)-based cartridge. Upon evaluating with the RNA samples of Omicron variant, the DMF RT-qPCR presented a sensitivity of 10 copies/μL and an amplification efficiency of 96.1%, capable for clinical diagnosis. When spiking with SARS-CoV-2 RNA (wildtype, Delta variant, or Omicron variant) and 18S rDNA, the clinical analog samples demonstrated accurate detection and discrimination of different SARS-CoV-2 strains in 49 min.

## 1. Introduction

Severe acute respiratory syndrome coronavirus 2 (SARS-CoV-2), a respiratory infectious agent, is responsible for the COVID-19 pandemic. As of 30 June 2023, there have been more than 767.5 million confirmed human cases of COVID-19 worldwide, including over 6.94 million deaths [1]. Despite the fact that more than 13.4 billion doses of vaccines have been administered and the additional boosters have been available [2], the infection rates were high due to the emergence of different variants of SARS-CoV-2, characterized by numerous mutations in the spike protein and across the genome [3,4]. Of all the different variants, the Delta variant, represented by lineage 1.617.2, was identified in late 2020 and quickly spread around the world, becoming the most virulent and dominant version of the coronavirus [5]. In November 2021, the Omicron variant (lineage B.1.1.529) was detected and the reported cases soon began to surge and multiply in other countries [6]. A subvariants of Omicron (XBB.1.5) then became the dominant variant globally due to its high transmissibility and potential immune evasion compared to earlier variants [7,8]. However, the full impact of the Omicron variant on disease severity and vaccine effectiveness is still under investigation [9,10]. 

The rapid and accurate diagnosis of SARS-CoV-2 and its important variants is pivotal to monitoring the spread, assessing the effectiveness of vaccines and therapy, and adjusting interventions during the pandemic [11]. Common diagnostic methods include serological tests that detect the presence of antibodies, IgM and IgG, in blood samples produced by the immune system in response to viral infection, and antigen tests that detect specific viral proteins in nasopharyngeal or oropharyngeal specimens [12,13,14]. However, serological tests are generally not used for the early diagnosis of active infection, but only to help assess whether an individual has been exposed to the virus [15]. While antigen testing is a rapid and less costly method, it is typically less sensitive and prone to false negatives in the early stages of infection when viral loads are low or when variant-specific antibodies are not available [16]. In contrast, reverse transcription quantitative polymerase chain reaction (RT-qPCR) has high sensitivity and specificity for the accurate detection of the viral RNA, and hence is the “gold standard” for the clinical diagnosis of SARS-CoV-2 [17,18]. By detecting multiple target genes in a single reaction, multiplex RT-qPCR offers high throughput with reduced reagents and fewer pipetting steps [19]. With proper primer–probe sets targeting unique deletions of SARS-CoV-2 variants, multiplex RT-qPCR has been proven to be a powerful tool for the identification and discrimination of wildtype, Delta variant, and Omicron variant [20,21].

RT-qPCR has been widely adapted for the point-of-care (POC) testing of SARS-CoV-2 outside of laboratory settings with a reduced sample consumption and short turnaround time [22,23,24,25,26]. Although multiplex RT-qPCR using multiple fluorescence-specific probes can improve the assay accuracy [27], the demand of POC’s portability could possibly compromise its optical functionalities and multiplexity. In addition, the potential temperature fluctuation and operation error in the scenario of on-site POC testing could cause undesired amplification products and PCR bias due to the complicated primer–probe sets [28,29,30]. Therefore, detecting multiple target genes of SARS-CoV-2 on a POC device is rare, not to mention discriminating its circulating variants. 

Here, we demonstrate a digital microfluidic (DMF) RT-qPCR cartridge for detecting and discriminating multiple SARS-CoV-2 strains, including wildtype, Delta variant, and Omicron variant. The study was built on our experiences on assay developments with proper primer–probe sets [20,21] and droplet manipulations on a DMF cartridge [31,32,33,34,35]. Different from typical multiplex PCR using different primer–probe sets in a single reaction, our multiplex scheme relies on concurrent singleplex reactions in multiple droplets each having its own primer–probe set. In other words, different primer–probe sets for wildtype, Delta variant, and Omicron variant, are mixed with the sample in separate droplets for RT-qPCR on the same DMF cartridge at the same time. This paradigm would minimize PCR bias that results from the competition among different primer–probe sets and eliminate the requirement of multiple fluorescence filters, which provides accurate and sensitive diagnostic results with a simple and compact system for RT-qPCR testing. The amplification efficiency and limit of detection (LoD) of the DMF RT-qPCR cartridge were evaluated using the Omicron variant RNA and compared with off-chip results from a benchtop qPCR instrument. The capability of detection and discrimination of SARS-CoV-2 variants was demonstrated using clinical analog samples.

## 2. Materials and Methods

### 2.1. DMF Cartridge

**Figure 1:**
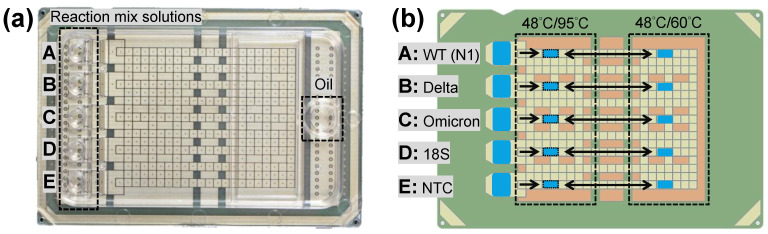
Digital microfluidic (DMF) cartridge for SARS-CoV-2 variants detection by RT-qPCR. (**a**) The top PC plate had five inlets for reaction mix solutions and an inlet for oil; the bottom PCB plate contained electrodes. (**b**) The five droplets generated from the reservoirs (indicated with rightwards arrows) underwent RT at 48 °C and then qPCR by shuttling between the 95 °C and 60 °C temperature zones (indicated with left right arrows) for the detection of SARS-CoV-2 wildtype (droplet A), Delta variant (droplet B), Omicron variant (droplet C), human 18S rRNA (droplet D) genes, and NTC (droplet E).

RT-qPCR was performed in assembled DMF cartridges (6 cm × 4 cm, Figure 1a) for SARS-CoV-2 variants diagnosis. Each cartridge was composed of a transparent top plate made of polycarbonate (PC) and a printed circuit board (PCB)-based bottom plate following the fabrication [35]. Briefly, the inner surface of the PC top plate was coated with a conductive polymer and a fluorinated hydrophobic layer. Five independent inlets and reservoirs (A to E) were designed for loading RT-qPCR reaction mix solutions, whereas an oil inlet was for the environmental fluid hexadecane. The PCB bottom plate held patterned copper electrodes that were covered with a polyimide film and a fluorinated hydrophobic layer. The reservoir electrodes were used for droplet generation, and a total of 274 square driving electrodes (1.5 mm × 1.5 mm) were used for droplet actuation. With proper generating procedures, the volume of the droplets, covering two driving electrodes (1.5 mm × 3 mm) between top and bottom plates with a gap height of 340 μm, was approximately 1.5 μL in this study.

The DMF cartridge was controlled by the electric, thermal, and optical modules reported previously [35]. Briefly, the electric module provided driving signals for droplet actuation; the thermal module adjusted and maintained the temperature of the two temperature zones of the cartridge (Figure 1b) for RT and PCR; the optical module obtained the fluorescent images for qPCR. To perform multiplex RT-qPCR on the DMF cartridge, the five droplets A to E were designed for detecting different target genes using corresponding primer–probe sets: SARS-CoV-2 wildtype (droplet A), Delta variant (droplet B), Omicron variant (droplet C), human 18S ribosomal RNA (rRNA, droplet D) genes, and all the above genes for no template control (NTC, droplet E), as shown in Figure 1b.

### 2.2. Reagents and Chemicals

Synthetic SARS-CoV-2 RNA controls (wildtype, part number: 103512; Delta, part number: 104539; Omicron, part number: 105857) with the concentration of 1,000,000 copies/μL were from Twist Bioscience (South San Francisco, CA, USA) and used as stock solutions. The human 18S ribosomal rDNA template was generously provided by our collaborator in the Veterinary Diagnostic Laboratory at Kansas State University (Manhattan, KS, USA). The 18S rDNA internal control template was extracted from transformed *E. coli* cells carrying the plasmid with target sequence to prepare the stock solution at the concentration of 3.72 ng/μL. The IDTE pH 7.5 (1× TE buffer) from Integrated DNA Technologies (IDT, Coralville, IA, USA) was used for RNA controls and 18S rDNA dilutions. The primer–probe sets used in this study are listed in Table S1. The RT-qPCR primer–probe set for N1 (2019-nCoV_N1-F, 2019-nCoV_N1-R, 2019-nCoV_N1-P) followed the panel reported by the U.S. Centers for Disease Control and Prevention (CDC) [36]; the primer–probe sets for Delta (SARS2-dF, SARS2-dR, SARS2-dPr, SARS2-wPr) and Omicron (OmN-F, OmN-R, OmNm-Pr, OmNw-Pr) variants were designed from a collection of 660,035 SARS-CoV-2 full- or near-full genomes, including 169,454 Delta variant and 24,202 Omicron variant strains [21]; the primer–probe set for the rDNA that codes for human 18S rRNA gene (18S-F, 18S-R, 18S-Pr), a conserved housekeeping gene, was referred to in a previous study [37]. All the primer–probe sets were synthesized from IDT. The 4× TaqPath^TM^ 1-Step Multiplex Master Mix for RT-qPCR in gene expression and quantitative analysis was produced by Applied Biosystems/ThermoFisher Scientific (Waltham, MA, USA). The surfactant, Tween-20 from Sigma-Aldrich (St. Louis, MO, USA), was supplemented in all solutions for droplet actuation [35,38,39]. The environmental fluid, hexadecane (99%), was from ThermoFisher Scientific [35,38].

### 2.3. RT-qPCR Reaction and Protocol

Five RT-qPCR reaction mix solutions are listed in Table S2. Other than the primer–probe set with its own optimized concentration, the final 20 μL solution also contained 5 μL of 4× TaqPath^TM^ 1-Step Multiplex Master Mix, 5 μL of nuclease-free water with 0.4% (*v/v*) Tween-20, and 5 μL of a sample except the NTC control. Based on the instruction of TaqPath^TM^ 1-Step Multiplex Master Mix, our RT-qPCR protocol followed a reverse transcription step at 48 °C for 10 min, a polymerase activation step at 95 °C for 2 min, and 45 cycles of denaturation at 95 °C for 3 s and annealing/extension at 60 °C for 20 s. All the target genes were recognized by the FAM-conjugated probes. It is noteworthy that the primer–probe set for Delta variant was designed to recognize the unique 6-nt deletion in the S gene (∆157–158 aa deletion), whereas the primer–probe set for Omicron was designed to recognize the unique 9-nt deletion in the N gene (∆31–33 aa deletion) [20,21]. To assure the probe specificity and eliminate false positives, an additional competing sequence of wildtype probe without fluorescent dye was designed and specifically supplemented in our Delta or Omicron primer–probe set. As listed in Tables S1 and S2, SARS-w-Pr probe was added in the Delta primer–probe set as a competing wildtype probe that prevents the Delta variant probe (SARS2-dPr) from binding to the non-Delta wildtype amplicons and generating a false-positive fluorescence signal. Similarly, the OmNw-Pr competing wildtype probe was used to bind to non-Omicron wildtype amplicons, eliminating false positive by non-specific binding of the Omicron variant probe (OmNm-Pr). The final concentrations of the variant and wildtype probes in the reaction mix solution were optimized. 

**Figure 2:**
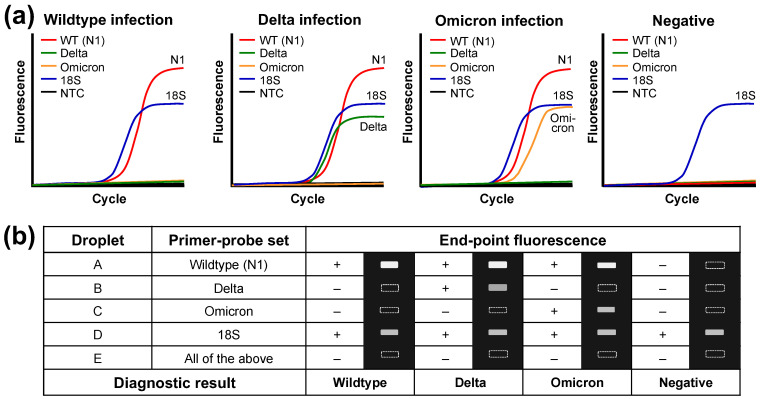
Four possible valid diagnostic results, wildtype, Delta, and Omicron positive and negative, from the DMF RT-qPCR. (**a**) Demonstrative amplification curves for different infection cases. (**b**) Representative end-point fluorescence signals (+/–) and images of the five droplets A–E for different infection cases.

Figure 2 shows the expected results of the four valid diagnostic results from the DMF cartridge. The amplification curves showing the fluorescence intensity against the PCR cycle number in Figure 2a can be used to confirm the infection of SARS-CoV-2 and discriminate the infection of wildtype, Delta variant, and Omicron variant strains. The threshold cycle (Ct) value obtained from the curve can be used to quantify the virus concentration. If only qualitative detection is required, the end-point fluorescence image will be used to discriminate the infections from different strains as shown in Figure 2b. Basically, droplet A determines whether the sample contains SARS-CoV-2 strains; droplet B and C detect and discriminate Delta and Omicron variants, respectively; droplet D confirms the valid human sample by 18S rRNA as an internal control to eliminate false negative; droplet E is for NTC to eliminate false positive from contamination and non-specific amplification. 

As listed in Table S2, we prepared different clinical analog samples (5 μL) representing wildtype, Delta variant, and Omicron variant infection cases by mixing 2.5 μL of SARS-CoV-2 RNA (80 copies/μL) and 2.5 μL of 18S rDNA (7.44 pg/μL). Hence, for all the clinical analog samples, including wildtype, Delta variant, and Omicron variant infections, the final SARS-CoV-2 RNA concentration was 10 copies/μL, and the final 18S rDNA concentration was 0.93 pg/μL in the 20 μL reaction mix solutions.

### 2.4. Data Analysis

During thermal cycling, the fluorescence intensity of a specific droplet was recorded along the cycles by measuring the average grayscale level per pixel within the droplet region of the corresponding images using ImageJ software (v. 1.51j8, National Institutes of Health, Bethesda, MD, USA) [40]. The baseline and the threshold signal level were determined automatically by LinRegPCR (2020.0, Dept. Medical Biology Amsterdam UMC, Amsterdam, The Netherlands) [41], which is a standalone program for analyzing raw fluorescence data and estimating the exponential phase by setting the window-of-linearity. The fluorescence results were expressed as delta fluorescence intensity (ΔFI) and plotted against PCR cycle number. By converting ΔFI to the logarithmic scale, the Ct value was determined for quantification analysis. The amplification efficiency of the PCR reaction was evaluated by conducting serial-dilution experiment, where the slope of the standard curve can be translated into efficiency by the following equation:

$$Efficiency=10^{\left(-\frac{1}{slope}\right)}-1$$

The acceptable PCR efficiency is generally considered to be within 90–110%, and 100% denotes the most optimized efficiency in PCR amplifications [42,43]. Note that the criterion for a sample to be considered positive is based on whether the measured fluorescence data can be analyzed for a reasonable Ct value, some of which show a slight increase in fluorescence intensity but cannot pass the background signal level and were thus regarded as background fluctuations.

## 3. Results

### 3.1. DMF RT-qPCR Performance 

With the previously confirmed high consistency in droplet volume, temperature uniformity, linearity of fluorescence intensity, and qPCR efficiency of the DMF cartridge [35], we first investigated the RT-qPCR performance with the SARS-CoV-2 Omicron variant RNA sample without 18S rDNA and the OmNw-Pr competing wildtype probe (Table S3). The stock solution of the Omicron RNA control with the concentration of 1,000,000 copies/μL from the vendor was serially diluted with TE buffer to obtain concentrations from 100,000 copies/µL to 10 copies/µL. A volume of 5 µL of Omicron RNA template solution was mixed into the reaction mix solution (final volume 20 µL, Table S3), which resulted in the final template concentrations from 25,000 copies/µL to 2.5 copies/µL. A volume of 7 µL of each prepared reaction mix solution (volume 20 µL, Table S3) was loaded into one of the reservoirs on the DMF cartridge for testing. Five droplets (each volume 1.5 µL) were simultaneously generated from the reservoirs (each volume 7 µL), driven onto the right temperature zone (Figure 1b), and kept at 48 °C for 10 min to perform RT of the Omicron variant RNA. When the droplets were positioned on the right temperature zone (48 °C) for the RT process, the left temperature zone was heated and maintained at 95 °C. After the RT step, the droplets were driven to the left temperature zone at 95 °C for polymerase activation for 2 min. Meanwhile, the right temperature zone was heated to 60 °C to prepare for the required annealing/extension temperature. Droplets were then shuttled between the two temperature zones to perform thermal cycling for qPCR amplification. At the annealing/extension step of each thermal cycle, a fluorescence image of all the droplets on the right temperature zone was captured for analysis. With a temperature ramp rate of 2.7 °C/s determined by the droplet moving velocity and the distance between two temperature zones on the DMF cartridge, the entire RT-qPCR procedure was completed within 49 min. 

**Figure 3:**
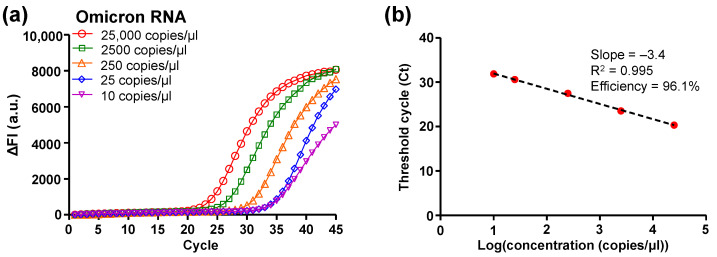
RT-qPCR of serially diluted SARS-CoV-2 Omicron variant RNA on the DMF cartridge. (**a**) RT-qPCR amplification curves of delta fluorescence intensity (ΔFI) against cycle number for various template concentrations. (**b**) Standard curve and efficiency on-chip.

The amplification efficiency and limit of detection (LoD) of the DMF RT-qPCR was evaluated based on the amplification curves (Figure 3a) and standard curve (Figure 3b) plotted from the captured fluorescence images. As the amplification proceeded, the higher concentration of the cDNA template corresponded to an earlier rise in the ΔFI curve in Figure 3a. The results showed that the DMF cartridge was able to detect Omicron variant RNA with a concentration of 10 copies/µL, which corresponded to 15 copies of RNA per reaction in the manipulated droplets (volume 1.5 µL) with the Omicron primer–probe set. The Ct values were plotted against the logarithm of the Omicron variant RNA concentration for evaluating the performance of the DMF cartridge, as shown in Figure 3b. The linear regression of the standard curve showed the slope of –3.4 and the correlation coefficient (R^2^) of 0.995. The qPCR efficiency was calculated as 96.1%, which is within the range of acceptable values (i.e., 90–110%). In Figure 3b, each data point was averaged from three replicates that showed a 100% positive percent agreement. Hence, the LoD was 10 copies/µL for DMF RT-qPCR with Omicron variant RNA, which is sufficient for clinical applications [20,21]. For the samples with a smaller concentration, e.g., 5 copies/µL and 2.5 copies/µL, we tested 10 replicates but not all the replicates showed significant amplification and a valid Ct value. The positive percent agreement was 50% (e.g., 5/10) for the concentration of 5 copies/µL (equivalent 7.5 copies per reaction) and 10% (e.g., 1/10) for 2.5 copies/µL (equivalent 3.75 copies per reaction). 

In addition to on-chip amplification, we also performed off-chip RT-qPCR using a benchtop qPCR instrument, LightCycler II (Roche Diagnostics, Indianapolis, IN, USA) for sample validation and data comparison. Basically, the RT-qPCR reaction mix solutions were prepared as previously described (Table S3) and the thermal cycling profile was set the same as that used for on-chip testing except a faster ramp rate at 20 °C/s. A volume of 10 µL of the prepared RT-qPCR reaction solutions (volume 20 µL, Table S3) was used for all off-chip amplifications. The on-chip amplification curves (Figure 3a) were comparable to those of off-chip ones (Figure S1). On-chip data showed better PCR efficiency, whereas off-chip ones showed a lower LoD. 

### 3.2. Clinical Analog Sample Diagnosis

**Figure 4:**
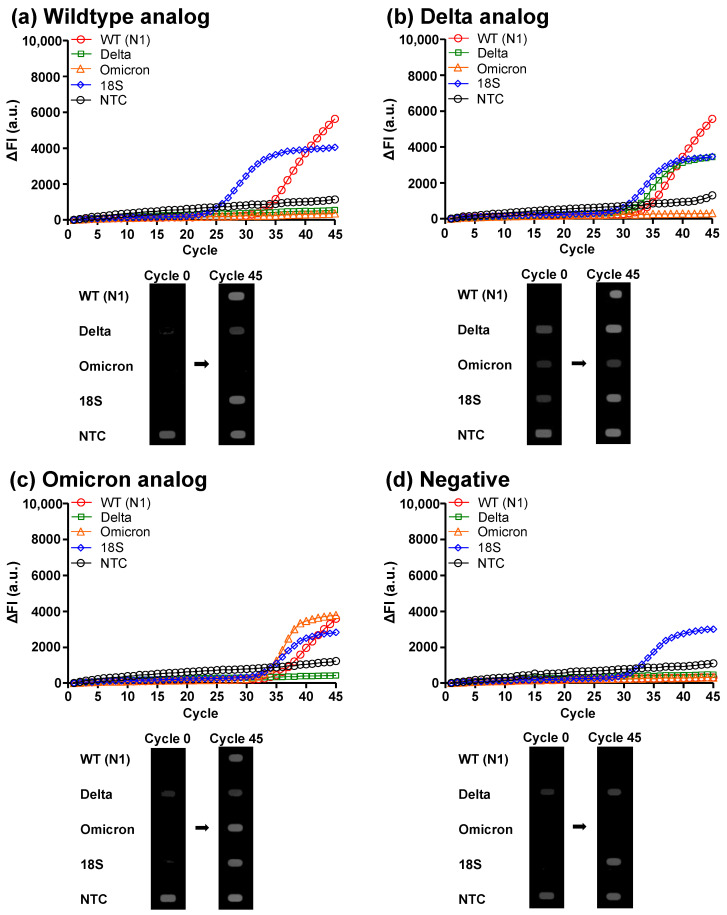
Demonstration of detection and discrimination of SARS-CoV-2 wildtype, Delta variant, and Omicron variant on the DMF cartridge. Amplification curves and initial (cycle 0) and end-point (cycle 45) fluorescence images of RT-qPCR for clinical analog samples representing (**a**) wildtype infection, (**b**) Delta variant infection, (**c**) Omicron variant infection, and (**d**) no infection.

After evaluating the RT-qPCR performance on the DMF cartridge, we prepared the clinical analog samples carrying the wildtype, Delta variant, or Omicron variant RNAs of SARS-CoV-2 with the final RNA concentration of 10 copies/µL (Table S2) for on-chip detection and discrimination of different SARS-CoV-2 strains. The amplification curves and the initial (cycle 0) and end-point (cycle 45) fluorescence images of the thermal cycles are shown in Figure 4 and Figure S2. For the wildtype case (Figure 4a), N1 and 18S curves showed a significant increase in fluorescence intensity, and the N1 and 18S droplets were brighter after the 45 PCR cycles. For the Delta case (Figure 4b), in addition to N1 and 18S genes, the Delta gene showed a significant increase in fluorescence, while the Omicron gene remained dark. Accordingly, the N1, Delta, and 18S droplets became bright in the fluorescence images for the Delta case. In contrast, for the Omicron case (Figure 4c), the Omicron gene showed an elevation in fluorescence, while the Delta gene did not. As a result, the N1, Omicron, and 18S droplets turned bright in the Omicron case. For the no infection case (Figure 4d), only the 18S gene showed an increase in fluorescence intensity, and only the 18S droplet was bright in the end-point fluorescence image. These results verified that the DMF RT-qPCR system was capable of detecting SARS-CoV-2 infections and discriminating wildtype, Delta variant, and Omicron variant. On the other hand, we noticed that the initial fluorescence signal (cycle 0) of the NTC in the droplet E was higher than other droplets because it contained all the fluorescent probes and the higher total probe concentration led to a stronger background fluorescence signal. The same situation was also observed in the raw data obtained from the off-chip experiment using the LightCycler instrument. 

## 4. Discussion

Different from our previous work focusing on on-chip qPCR for SARS-CoV-2 N1 and N2 genes detection [35], in this paper, we developed on-chip DMF RT-qPCR for the detection and discrimination of SARS-CoV-2 variants. Compared to previous work, the RT step was added to the protocol, which demonstrated the flexibility of droplet and temperature control of the DMF system. Instead of using probes with different fluorescent dyes to identify multiple nucleic acid targets (i.e., N1, Delta, Omicron, and 18S genes) in a single reaction, DMF RT-qPCR cartridge used multiple droplets that contained singleplex target-specific primer–probe sets to perform multiplex detection. As there was only one primer–probe set in each droplet, the competition among different primer–probe sets was minimized to avoid PCR bias. Additionally, as different targets were identified using the same fluorescent dye (i.e., FAM), multiple fluorescence filters were not required, which rendered a simple and compact system suitable for POC testing. 

We demonstrated the high RT-qPCR efficiency on the DMF cartridge when amplifying serially diluted SARS-CoV-2 Omicron variant RNA. The LoD was 10 copies/μL using 1.5 μL droplets, which is sufficient for clinical applications [20,21]. The positive percent agreement for samples with 5 copies/μL (12.5 copies/droplet) and 2.5 copies/μL (3.75 copies/droplet) RNA was 50% and 10%, respectively, on the DMF cartridge due to the small droplet volume. A detection of lower concentrations with a 100% positive percent agreement could be alternatively achieved by increasing the volume of the reaction droplet.

Unique deletions in N gene and S gene were used to identify Delta (∆157–158) and Omicron (∆31–33) variants, respectively, as mentioned above [20,21]. Because the deletion length is only 6 nt (Delta) or 9 nt (Omicron), without a wildtype probe in the reaction mix solution, a portion of the variant probe could non-specifically bind to the non-variant wildtype amplicon and cause a false positive fluorescence signal which was experimentally confirmed. The wildtype probes did not carry fluorescent dye. With the sequence-specific variant and wildtype probes (Table S1) designed in the same gene region for competitive hybridization to the template, high specificity and explicit results for both positive (bright for variant) and negative (dark for wildtype) samples were obtained. In contrast, although having been adapted for POC testing, the detection of variants using target failure RT-qPCR (e.g., S gene ∆69–70 target failure for Omicron variant detection) [27,44] is implicit and prone to be false-positive.

Compared to DMF RT-qPCR, the off-chip assay with the LightCycler instrument provided a faster temperature ramp rate at 20 °C/s. However, because we did not heat and cool the entire DMF cartridge, the demonstrated temperature ramp rate (2.7 °C/s) can be increased in the future by decreasing the distance between temperature zones and increasing the droplet velocity. Hence, the demonstrated total on-chip RT-qPCR time of approximately 49 min can be reduced. In addition, by optimizing the reaction mix with higher polymerase and primer concentrations, the required time for denaturation and annealing/extension can be further reduced, even for achieving extreme PCR in the future [45]. 

Using the fluorescence images of all five droplets in each PCR cycle, we noticed a slight decrease in droplet size after 45 thermal cycles with the tested environmental fluid hexadecane. The decrease in droplet size resulted in a small increase in fluorescent signal but did not affect the PCR detection results when analyzed with the LinRegPCR program. Different environmental fluids can be tested in the future to eliminate evaporation. 

Although on-chip viral lysis and RNA extraction were not investigated in this study, lysis and magnetic-bead-based extraction steps can be integrated on the current DMF cartridge with some minor adjustment in the control system [33,38,39]. Alternatively, our current on-chip procedure is ready for the extraction-free SARS-CoV-2 RT-qPCR protocol developed during the COVID-19 pandemic to cope with the shortages of RNA extraction kits [46,47,48,49,50]. Briefly, after direct viral inactivation and lysis by heat in the viral transport media, the sample can be added in the RT-qPCR reaction mix solutions and loaded to the DMF cartridge for RT-qPCR detection and discrimination. 

## 5. Conclusions

We presented POC testing for the detection and discrimination of SARS-CoV-2 wildtype, Delta variant, and Omicron variant infections based on DMF RT-qPCR. Multiple nucleic acid targets, N1, Delta, Omicron, and 18S genes, were amplified and identified in different droplets manipulated on individual tracks, which allowed for multiplex detection with a minimized PCR bias caused by competition among primer–probe sets using a simple and compact setup. Requiring less sample volume, the amplification efficiency and sensitivity of the DMF RT-qPCR cartridge were comparable to those obtained from off-chip RT-qPCR using the LightCycler instrument. The capability of the DMF RT-qPCR cartridge to detect and discriminate SARS-CoV-2 wildtype, Delta variant, and Omicron variant strains was demonstrated.

## Data Availability

The data presented in this study are available upon request from the corresponding author.

## Supplementary Materials

The following supporting information can be downloaded at: https://www.mdpi.com/article/10.3390/mi14081627/s1, Table S1: Primers and probes used in this study; Table S2: RT-qPCR reaction mix solutions for detection and discrimination of SARS-CoV-2 wildtype, Delta variant, and Omicron variant; Table S3: RT-qPCR reaction mix solutions for detection of SARS-CoV-2 Omicron variant; Figure S1: Off-chip RT-qPCR of serially diluted SARS-CoV-2 Omicron variant RNA. (a) RT-qPCR amplification curves for various concentrations. (b) Standard curve and RT-qPCR efficiency off-chip; Figure S2: The uncropped initial (cycle 0) and end-point (cycle 45) fluorescence images of RT-qPCR for clinical analog samples representing (a) wildtype infection, (b) Delta variant infection, (c) Omicron variant infection, and (d) no infection; Figure S3: Off-chip RT-qPCR verification of SARS-CoV-2 wildtype RNA detection and Delta and Omicron variants discrimination. Amplification curves for clinical analog samples representing (a) wildtype infection, (b) Delta variant infection, (c) Omicron variant infection, and (d) no infection.

## References

[1] WHO Coronavirus (COVID-19) Dashboard. https://covid19.who.int/.

[2] Total COVID-19 Vaccine Doses Administered. https://ourworldindata.org/grapher/cumulative-covid-vaccinations.

[3] What COVID-19 Variants Are Going Around in June 2023?. https://www.nebraskamed.com/COVID/what-covid-19-variants-are-going-around.

[4] Magazine N., Zhang T., Wu Y., McGee M.C., Veggiani G., Huang W. (2022). Mutations and Evolution of the SARS-CoV-2 Spike Protein. Viruses.

[5] Mlcochova P., Kemp S.A., Dhar M.S., Papa G., Meng B., Ferreira I., Datir R., Collier D.A., Albecka A., Singh S. (2021). SARS-CoV-2 B.1.617.2 Delta variant replication and immune evasion. Nature.

[6] Khandia R., Singhal S., Alqahtani T., Kamal M.A., El-Shall N.A., Nainu F., Desingu P.A., Dhama K. (2022). Emergence of SARS-CoV-2 Omicron (B.1.1.529) variant, salient features, high global health concerns and strategies to counter it amid ongoing COVID-19 pandemic. Environ. Res..

[7] Carabelli A.M., Peacock T.P., Thorne L.G., Harvey W.T., Hughes J., Consortium C.-G.U., Peacock S.J., Barclay W.S., de Silva T.I., Towers G.J. (2023). SARS-CoV-2 variant biology: Immune escape, transmission and fitness. Nat. Rev. Microbiol..

[8] Vogel L. (2023). What to know about Omicron XBB.1.5. Can. Med. Assoc. J..

[9] Andrews N., Stowe J., Kirsebom F., Toffa S., Rickeard T., Gallagher E., Gower C., Kall M., Groves N., O’Connell A.M. (2022). Covid-19 Vaccine Effectiveness against the Omicron (B.1.1.529) Variant. N. Engl. J. Med..

[10] Lau J.J., Cheng S.M.S., Leung K., Lee C.K., Hachim A., Tsang L.C.H., Yam K.W.H., Chaothai S., Kwan K.K.H., Chai Z.Y.H. (2023). Real-world COVID-19 vaccine effectiveness against the Omicron BA.2 variant in a SARS-CoV-2 infection-naive population. Nat. Med..

[11] Cocherie T., Zafilaza K., Leducq V., Marot S., Calvez V., Marcelin A.G., Todesco E. (2022). Epidemiology and Characteristics of SARS-CoV-2 Variants of Concern: The Impacts of the Spike Mutations. Microorganisms.

[12] Kevadiya B.D., Machhi J., Herskovitz J., Oleynikov M.D., Blomberg W.R., Bajwa N., Soni D., Das S., Hasan M., Patel M. (2021). Diagnostics for SARS-CoV-2 infections. Nat. Mater..

[13] Renard N., Daniel S., Cayet N., Pecquet M., Raymond F., Pons S., Lupo J., Tourneur C., Pretis C., Gerez G. (2021). Performance Characteristics of the Vidas SARS-CoV-2 IgM and IgG Serological Assays. J. Clin. Microbiol..

[14] Korenkov M., Poopalasingam N., Madler M., Vanshylla K., Eggeling R., Wirtz M., Fish I., Dewald F., Gieselmann L., Lehmann C. (2021). Evaluation of a Rapid Antigen Test to Detect SARS-CoV-2 Infection and Identify Potentially Infectious Individuals. J. Clin. Microbiol..

[15] Sidiq Z., Hanif M., Dwivedi K.K., Chopra K.K. (2020). Benefits and limitations of serological assays in COVID-19 infection. Indian J. Tuberc..

[16] Robinson M.L., Mirza A., Gallagher N., Boudreau A., Garcia Jacinto L., Yu T., Norton J., Luo C.H., Conte A., Zhou R. (2022). Limitations of Molecular and Antigen Test Performance for SARS-CoV-2 in Symptomatic and Asymptomatic COVID-19 Contacts. J. Clin. Microbiol..

[17] Green D.A., Zucker J., Westblade L.F., Whittier S., Rennert H., Velu P., Craney A., Cushing M., Liu D., Sobieszczyk M.E. (2020). Clinical Performance of SARS-CoV-2 Molecular Tests. J. Clin. Microbiol..

[18] Gdoura M., Abouda I., Mrad M., Ben Dhifallah I., Belaiba Z., Fares W., Chouikha A., Khedhiri M., Layouni K., Touzi H. (2022). SARS-CoV2 RT-PCR assays: In vitro comparison of 4 WHO approved protocols on clinical specimens and its implications for real laboratory practice through variant emergence. Virol. J..

[19] Kudo E., Israelow B., Vogels CB F., Lu P., Wyllie A.L., Tokuyama M., Venkataraman A., Brackney D.E., Ott I.M., Petrone M.E. (2020). Detection of SARS-CoV-2 RNA by multiplex RT-qPCR. PLoS Biol..

[20] Hamill V., Noll L., Lu N., Tsui WN T., Porter E.P., Gray M., Sebhatu T., Goerl K., Brown S., Palinski R. (2022). Molecular detection of SARS-CoV-2 strains and differentiation of Delta variant strains. Transbound. Emerg. Dis..

[21] Tsui WN T., Hamill V., Noll L., Lu N., Porter E.P., Harbidge D., Cox E., Richardson C., Gray M., Sebhatu T. (2022). Molecular detection of SARS-CoV-2 and differentiation of Omicron and Delta variant strains. Transbound. Emerg. Dis..

[22] Fernandes R.S., de Oliveira Silva J., Gomes K.B., Azevedo R.B., Townsend D.M., de Paula Sabino A., Branco de Barros A.L. (2022). Recent advances in point of care testing for COVID-19 detection. Biomed. Pharmacother..

[23] Valera E., Jankelow A., Lim J., Kindratenko V., Ganguli A., White K., Kumar J., Bashir R. (2021). COVID-19 Point-of-Care Diagnostics: Present and Future. ACS Nano.

[24] Kalia R., Kaila R., Kahar P., Khanna D. (2022). Laboratory and Point-of-Care Testing for COVID-19: A Review of Recent Developments. Cureus.

[25] Song Q., Sun X., Dai Z., Gao Y., Gong X., Zhou B., Wu J., Wen W. (2021). Point-of-care testing detection methods for COVID-19. Lab Chip.

[26] Yin B., Wan X., Sohan A., Lin X. (2022). Microfluidics-Based POCT for SARS-CoV-2 Diagnostics. Micromachines.

[27] Dakhave M., Gadekar S., Malekar A., Wankhede G. (2023). ‘CoviSwiftTM’: A point-of-care RT-PCR device for SARS-CoV-2 and its variant detection. J. Virol. Methods.

[28] Brownie J., Shawcross S., Theaker J., Whitcombe D., Ferrie R., Newton C., Little S. (1997). The elimination of primer-dimer accumulation in PCR. Nucleic Acids Res..

[29] Xie N.G., Wang M.X., Song P., Mao S., Wang Y., Yang Y., Luo J., Ren S., Zhang D.Y. (2022). Designing highly multiplex PCR primer sets with Simulated Annealing Design using Dimer Likelihood Estimation (SADDLE). Nat. Commun..

[30] Elnifro E.M., Ashshi A.M., Cooper R.J., Klapper P.E. (2000). Multiplex PCR: Optimization and application in diagnostic virology. Clin. Microbiol. Rev..

[31] Huang C.-Y., Shih P.-H., Tais P.-Y., Lee I.-C., Hsu H.-Y., Huang H.-Y., Fan S.-K., Hsu W. (2015). AMPFLUID: Aggregation magnified post-assay fluorescence for ultrasensitive immunodetection on digital microfluidics. Proc. IEEE.

[32] Chiang M.Y., Hsu Y.W., Hsieh H.Y., Chen S.Y., Fan S.K. (2016). Constructing 3D heterogeneous hydrogels from electrically manipulated prepolymer droplets and crosslinked microgels. Sci. Adv..

[33] Hung P.-Y., Jiang P.-S., Lee E.-F., Fan S.-K., Lu Y.-W. (2017). Genomic DNA extraction from whole blood using a digital microfluidic (DMF) platform with magnetic beads. Microsyst. Technol..

[34] Komatsu T., Tokeshi M., Fan S.-K. (2022). Determination of Blood Lithium-Ion Concentration via Digital Microfluidic Whole-Blood Separation and Preloaded Paper Sensors. Biosens. Bioelectron..

[35] Ho K.L., Liao H.Y., Liu H.M., Lu Y.W., Yeh P.K., Chang J.Y., Fan S.K. (2022). Digital Microfluidic qPCR Cartridge for SARS-CoV-2 Detection. Micromachines.

[36] Lu X., Wang L., Sakthivel S.K., Whitaker B., Murray J., Kamili S., Lynch B., Malapati L., Burke S.A., Harcourt J. (2020). US CDC Real-Time Reverse Transcription PCR Panel for Detection of Severe Acute Respiratory Syndrome Coronavirus 2. Emerg. Infect. Dis..

[37] Wang Y., Das A., Zheng W., Porter E., Xu L., Noll L., Liu X., Dodd K., Jia W., Bai J. (2020). Development and evaluation of multiplex real-time RT-PCR assays for the detection and differentiation of foot-and-mouth disease virus and Seneca Valley virus 1. Transbound. Emerg. Dis..

[38] Sista R., Hua Z., Thwar P., Sudarsan A., Srinivasan V., Eckhardt A., Pollack M., Pamula V. (2008). Development of a digital microfluidic platform for point of care testing. Lab Chip.

[39] Sista R.S., Eckhardt A.E., Srinivasan V., Pollack M.G., Palanki S., Pamula V.K. (2008). Heterogeneous immunoassays using magnetic beads on a digital microfluidic platform. Lab Chip.

[40] Schneider C.A., Rasband W.S., Eliceiri K.W. (2012). NIH Image to ImageJ: 25 years of image analysis. Nat. Methods.

[41] LinRegPCR. https://medischebiologie.nl/files/.

[42] Svec D., Tichopad A., Novosadova V., Pfaffl M.W., Kubista M. (2015). How good is a PCR efficiency estimate: Recommendations for precise and robust qPCR efficiency assessments. Biomol. Detect. Quantif..

[43] Kralik P., Ricchi M. (2017). A Basic Guide to Real Time PCR in Microbial Diagnostics: Definitions, Parameters, and Everything. Front. Microbiol..

[44] Brown K.A., Gubbay J., Hopkins J., Patel S., Buchan S.A., Daneman N., Goneau L.W. (2021). S-Gene Target Failure as a Marker of Variant B.1.1.7 Among SARS-CoV-2 Isolates in the Greater Toronto Area, December 2020 to March 2021. JAMA.

[45] Farrar J.S., Wittwer C.T. (2015). Extreme PCR: Efficient and specific DNA amplification in 15–60 seconds. Clin. Chem..

[46] Delgado-Diaz D.J., Sakthivel D., Nguyen HH T., Farrokzhad K., Hopper W., Narh C.A., Richards J.S. (2022). Strategies That Facilitate Extraction-Free SARS-CoV-2 Nucleic Acid Amplification Tests. Viruses.

[47] Byrnes S.A., Gallagher R., Steadman A., Bennett C., Rivera R., Ortega C., Motley S.T., Jain P., Weigl B.H., Connelly J.T. (2021). Multiplexed and Extraction-Free Amplification for Simplified SARS-CoV-2 RT-PCR Tests. Anal. Chem..

[48] Smyrlaki I., Ekman M., Lentini A., Rufino de Sousa N., Papanicolaou N., Vondracek M., Aarum J., Safari H., Muradrasoli S., Rothfuchs A.G. (2020). Massive and rapid COVID-19 testing is feasible by extraction-free SARS-CoV-2 RT-PCR. Nat. Commun..

[49] Hasan M.R., Mirza F., Al-Hail H., Sundararaju S., Xaba T., Iqbal M., Alhussain H., Yassine H.M., Perez-Lopez A., Tang P. (2020). Detection of SARS-CoV-2 RNA by direct RT-qPCR on nasopharyngeal specimens without extraction of viral RNA. PLoS ONE.

[50] Fomsgaard A.S., Rosenstierne M.W. (2020). An alternative workflow for molecular detection of SARS-CoV-2-escape from the NA extraction kit-shortage, Copenhagen, Denmark, March 2020. Eurosurveillance.

