# Supplementary material for: Digital Microfluidic Multiplex RT-qPCR for SARS-CoV-2 Detection and Variants Discrimination
Source: Micromachines (Basel). 2023 Aug 17;14(8):1627. doi: 10.3390/mi14081627 (PMC10456927; doi:10.3390/mi14081627)
Supplement: Supplementary file 1 [file micromachines-14-01627-s001.zip › micromachines-2514535-SI.pdf]

Supplementary information to

Digital Microfluidic RT-qPCR Cartridge for Detection of SARS-CoV-2 and Discrimination of Delta and Omicron Variants

Kuan-Lun Ho [1], Jing Ding [1], Jia-Shao Fan [2], Wai Ning Tiffany Tsui [3], Jianfa Bai [3], and Shih-Kang Fan [1,*]

[1] Department of Mechanical and Nuclear Engineering, Kansas State University, Manhattan, KS 66506, USA
[2] Department of Electrical and computer Engineering, Kansas State University, Manhattan, KS 66506, USA
[3] Department of Diagnostic Medicine/Pathobiology, Kansas State University, Manhattan, KS 66506, USA
* Correspondence: skfan@ksu.edu

Supplementary Information includes:

Table S1. Primers and probes used in this study.

Table S2. RT-qPCR reaction mix solutions for detection and discrimination of SARS-CoV-2 wildtype, Delta variant, and Omicron variant.

Table S3. RT-qPCR reaction mix solutions for detection of SARS-CoV-2 Omicron variant.

Figure S1. Off-chip RT-qPCR of serially diluted SARS-CoV-2 Omicron variant RNA. (a) RT-qPCR amplification curves for various concentrations. (b) Standard curve and RT-qPCR efficiency off-chip.

Figure S2. The uncropped initial (cycle 0) and end-point (cycle 45) fluorescence images of RT-qPCR for clinical analog samples representing (a) wildtype infection, (b) Delta variant infection, (c) Omicron variant infection, and (d) no infection.

Figure S3. Off-chip RT-qPCR verification of SARS-CoV-2 wildtype RNA detection and Delta and Omicron variants discrimination. Amplification curves for clinical analog samples representing (a) wildtype infection, (b) Delta variant infection, (c) Omicron variant infection, and (d) no infection.

Table S1. Primers and probes used in this study.

Primer/probe name	Sequence (5′-3′)	Amplicon size
2019-nCoV_N1-F	GACCCCAAAATCAGCGAAAT	72 bp
2019-nCoV_N1-R	TCTGGTTACTGCCAGTTGAATCTG	
2019-nCoV_N1-P	FAM-ACCCCGCATTACGTTTGGTGGACC	
SARS2-dF	CCACAAAAACAACAAAAGTTGG	78 bp for Delta<br>84 bp for non-Delta
SARS2-dR	TGAGAGACATATTCAAAAGTGCAA	
SARS2-dPr	FAM-ATAAACTCCACTTTCCA-MGB	
SARS2-wPr	AGAATAAACTCTGAACTCACTTTCCATCC	
OmN-F	GGACCCTCAGATTCAACTGG	86 bp for Omicron<br>95 bp for non-Omicron
OmN-R	GCAGTATTATTGGGTAAACCTTGG	
OmNm-Pr	FAM-ATCGCGCCCCACCATTCT	
OmNw-Pr	CGCCCCACTGCGTTCTCC	
18S-F	GGAGTATGGTTGCAAAGCTGA	100 bp
18S-R	GGTGAGGTTTCCCGTGTTG	
18S-Pr	FAM-AAGGAATTGACGGAAGGGCA	

Table S2. RT-qPCR reaction mix solutions for detection and discrimination of SARS-CoV-2 wildtype, Delta variant, and Omicron variant.

**Droplet (1.5 µl)**		A	B	C	D	E
**Target Gene**		N1	Delta	Omicron	18S	NTC
**RT-qPCR Component**		Volume (µl)				
TaqPath™ 1-Step RT-qPCR Master Mix (4X)		5	5	5	5	5
Nuclease free water w/ 0.4% (v/v) tween-20		5	5	5	5	5
Nuclease free water		3	2.5	2.25	3	0.75
N1	N1 Primer mix (10 µM)	1	0	0	0	1
	N1-P (10 µM, FAM)	1	0	0	0	1
Delta	SARS2-d Primer mix (10 µM)	0	1	0	0	1
	SARS2-dPr (10 µM, FAM)	0	0.5	0	0	0.5
	SARS2-wPr (15 µM)	0	1	0	0	1
Omicron	OmN Primer mix (10 µM)	0	0	1	0	1
	OmNm-Pr (10 µM, FAM)	0	0	0.75	0	0.75
	OmNw-Pr (15 µM)	0	0	1	0	1
18S	18S Primer mix (10 µM)	0	0	0	1	1
	18S-Pr (10 µM, FAM)	0	0	0	1	1
Sample	SARS-CoV-2 RNA (WT, Delta, or Omicron, 80 copies/µl)	2.5	2.5	2.5	2.5	0
	18S rDNA (7.44 pg/µl)	2.5	2.5	2.5	2.5	0
**Total volume**		**20**	**20**	**20**	**20**	**20**

Table S3. RT-qPCR reaction mix solutions for detection of SARS-CoV-2 Omicron variant.

RT-qPCR Component	Volume (μl)
TaqPath™ 1-Step RT-qPCR Master Mix (4X)	5
Nuclease free water w/ 0.4% (v/v) tween-20	5
Nuclease free water	3.25
OmN Primer mix (10 μM)	1
OmNm-Pr (10 μM, FAM)	0.75
Omicron RNA Template (100,000 copies/μl to 10 copies/μl)	5
**Total**	**20**

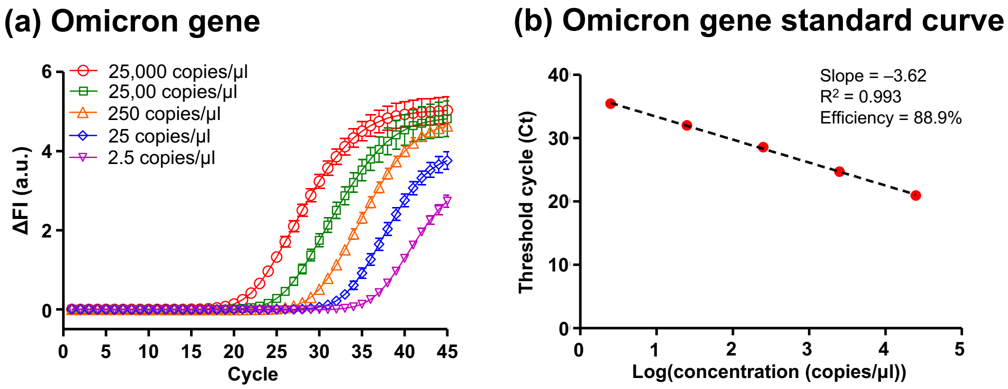

Figure S1. Off-chip RT-qPCR of serially diluted SARS-CoV-2 Omicron variant RNA. (a) RT-qPCR amplification curves for various concentrations. (b) Standard curve and RT-qPCR efficiency off-chip.

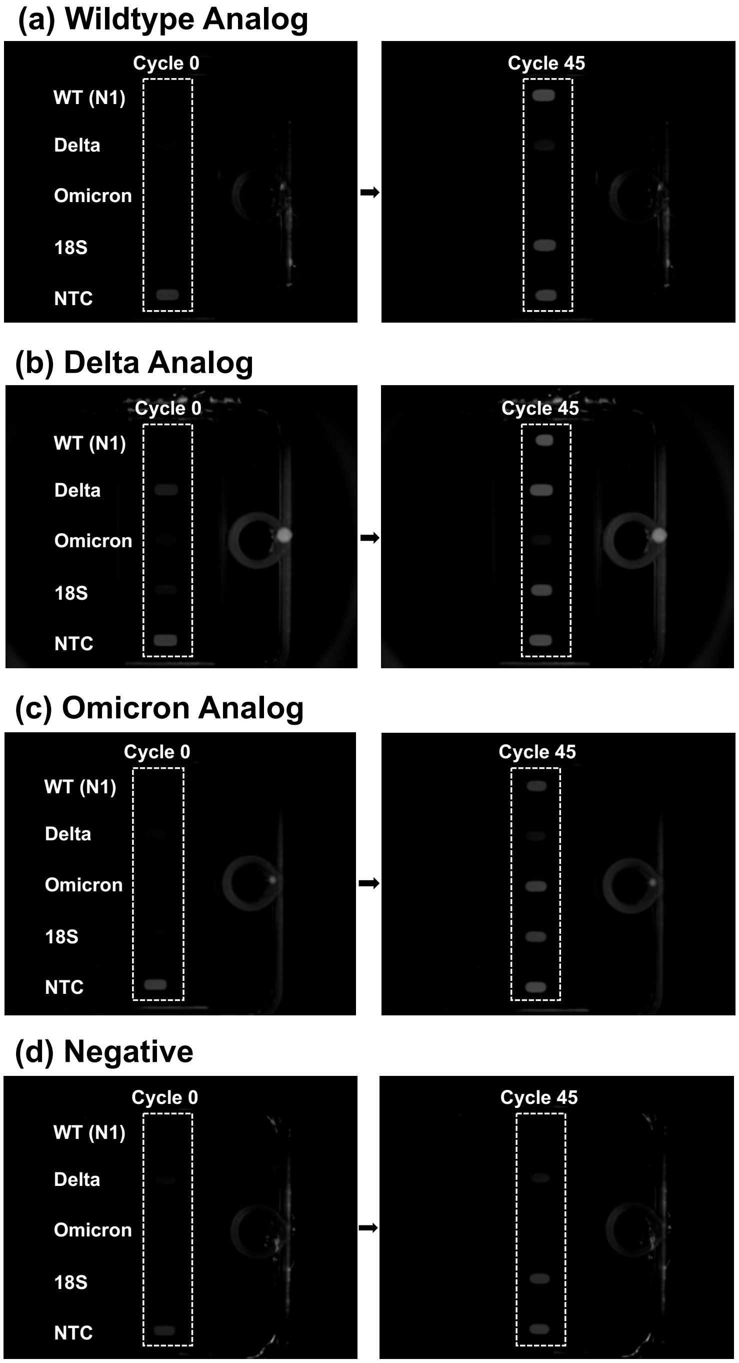

Figure S2. The uncropped initial (cycle 0) and end-point (cycle 45) fluorescence images of RT-qPCR for clinical analog samples representing (a) wildtype infection, (b) Delta variant infection, (c) Omicron variant infection, and (d) no infection.

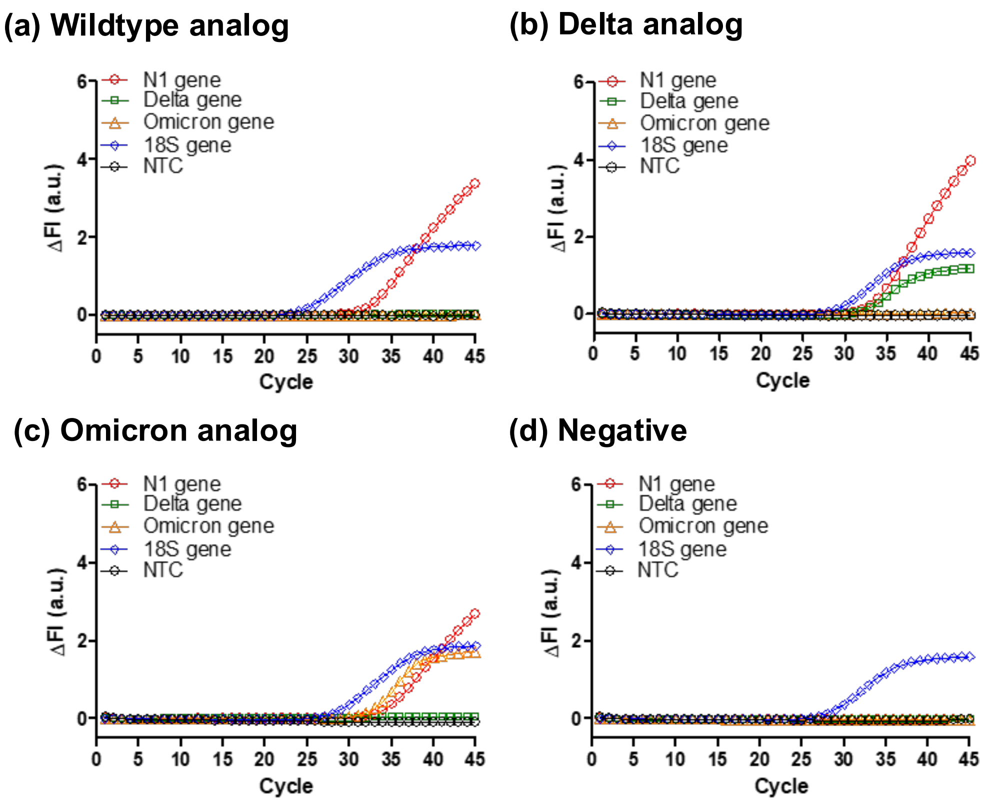

Figure S3. Off-chip RT-qPCR verification of SARS-CoV-2 wildtype RNA detection and Delta and Omicron variants discrimination. Amplification curves for clinical analog samples representing (a) wildtype infection, (b) Delta variant infection, (c) Omicron variant infection, and (d) no infection.
